# Type 2 Diabetes Mellitus and COVID-19: A Narrative Review

**DOI:** 10.3389/fendo.2021.609470

**Published:** 2021-03-31

**Authors:** Salvatore Corrao, Karen Pinelli, Martina Vacca, Massimo Raspanti, Christiano Argano

**Affiliations:** ^1^ Department of Internal Medicine, National Relevance and High Specialization Hospital Trust ARNAS Civico, Di Cristina, Benfratelli, Palermo, Italy; ^2^ Dipartimento di Promozione della Salute, Materno Infantile, Medicina Interna e Specialistica di Eccellenza "G. D'Alessandro", PROMISE, University of Palermo, Palermo, Italy

**Keywords:** COVID-19, SARS-CoV-2, coronavirus, diabetes, chronic conditions, review

## Abstract

The pandemic of coronavirus disease (COVID-19), caused by severe acute respiratory syndrome coronavirus 2 (SARS-CoV-2), has involved more than one hundred million individuals, including more than two million deaths. Diabetes represents one of the most prevalent chronic conditions worldwide and significantly increases the risk of hospitalization and death in COVID-19 patients. In this review, we discuss the prevalence, the pathophysiological mechanisms, and the outcomes of COVID-19 infection in people with diabetes. We propose a rationale for using drugs prescribed in patients with diabetes and some pragmatic clinical recommendations to deal with COVID-19 in this kind of patient.

## Introduction

In early December 2019, the first pneumonia cases of unknown origin were identified in Wuhan, the capital city of Hubei province. The novel pathogen was an enveloped RNA-beta-coronavirus-2 named Severe Acute Respiratory Syndrome Coronavirus 2 (SARS-CoV-2), with phylogenetic similarity SARS-CoV (1). Initially, the outbreak was reported starting from a zoonotic transmission in live animal and seafood market. It soon became apparent that efficient person-to-person transmission was also occurring (2). The disease has rapidly spread from Wuhan to other areas. By March 11, 2020, the WHO (World Health Organization) declared the pandemic status (3). Globally, up to February 3, 2021, there have been 103.201.340 confirmed cases of COVID-19, including 2.237.636 deaths, reported to COVID-19 Dashboard by the Center for Systems Science and Engineering (CSSE) at Johns Hopkins University.

Considering the rapid spread and high mortality rate of COVID-19, it is necessary to evaluate the possible risk factors affecting the progression of disease in COVID-19 patients (4).

The pandemic has involved millions of persons, and their related chronic conditions could have prognostic and therapeutic implications. One of the essential chronic conditions is, without any doubt, diabetes for its impact on hospitalization, mortality, and economic burden. To date, about half a billion people have diabetes worldwide, and the number will increase by 25% in 2030 and 51% in 2045 (5). The prevalence is estimated to be 9.3% (463 million people), rising to 10.2% by 2030 and 10.9% by 2045 (5). According to various studies, the prevalence of diabetes in COVID-19 patients ranged from 5% to 36%. Considering that diabetes is one of the most important comorbidities in SARS patients, it is necessary to clarify all the aspects concerning the links between the two conditions to offer the scientific and clinical community those elements useful to face this pandemic in the best possible way. An extensive search of SCOPUS, PubMed, and CENTRAL was performed using the following string “(SARS-Cov-2 OR COVID-19) AND (diabetes OR hyperglycemia)”. The hand-searching of principal generalist and infectious disease Journals was carried out as well. This review aimed to summarize the special aspects of COVID-19 infection in people with diabetes and the relation between diabetes and COVID-19 in epidemiology, pathophysiology, prognosis, and therapeutics to provide practical and clinical recommendations approaching diabetic patients during this pandemic.

## Prevalence of Diabetes and Its Clinical Severity in Patients With COVID-19

Emerging data suggest that COVID-19 is common in patients with diabetes, hypertension, and cardiovascular diseases (CVD), even if the prevalence rate changed in different studies and country-wise data (6). The rates of type 2 diabetes in subjects affected by SARS-CoV-2 vary, depending on the median age, the severity of illness, the location of the study population, and the method of testing.

**Table 1** shows a synthesis of various studies from an extensive search on bibliographic citation databases (7–16) that explored the prevalence of diabetes according to the type of study, population, sample size, age, sex, and prevalence of diabetes, hypertension, and obesity.

**Table 1:** Prevalence of type 2 diabetes, hypertension and cardiovascular disease in various studies on COVID-19 patients.

Authors	Type of study	Population	N of patients	Age (median)	Female (%)	Diabetes (%)	H.T.N. (%)	Obesity (%)
Liang W. et al. (7)	Retrospective study	Chinese	1590	NR	42.7	8.2	16.9	NR
Singh et al. (8)	Systematic Review	Chinese	2209	40-57	41.7	11.0	21.0	NR
Yang et al. (9)	Meta-analysis	Chinese	46.248	49.6	43.5	8.0	17.0	NR
Wu Z. et al. (10)	Retrospective study	Chinese	72314	NR	NR	7.3	6.0	NR
Richardson S. et al. (11)	Case Series	North American	5700	63.0	39.7	33.8	56.6	41.7
Petrilli et al. (12)	Cross-sectional analysis	North American	4103	52.0	49.5	15.0	24.0	30.1
Mandeep et al. (13)	Observational multicentric database	North American, European, Asian	8910	49.0	40.0	14.3	26.3	NR
Onder et al. (14)	Retrospective study	Italian	355	79.5	30.0	35.5	NR	NR
COVID-19	Registry	Italian	481	80.0	29.4	33.9	7.3	NR
Surveillance group (15)								
Grasselli et al. (16)	Retrospective case-series	Italian	1043	63.0	28.0	17.0	49.0	NR

NR, not reported.

Data show that the prevalence of diabetes and sex differences (with a higher prevalence of men than women), increases according to the increase of median age. A similar trend regarded hypertension. Current evidence indicates that fatality rates are higher in men than in women. A report on April 23, 2020, from the Italian National Institute of Health, shows that of 23.188 deaths from COVID-19 infection in Italy, approximately 70% were in men (17). Besides, our previously published data in elderly inpatients (18, 19) have shown a higher prevalence of diabetes in men than women. This evidence might explain the higher prevalence of diabetes in elderly patients affected by COVID-19. It could justify an exclusively epidemiological association between diabetes and COVID-19 and not the virus’s ability to affect diabetic patients specifically.

There are two other aspects to consider: how much does diabetes affect clinical severity? Since its relationship to, how does obesity? Some data might clarify these issues.

Firstly, U.S.A. Centers for Disease Control and Prevention reported data for laboratory-confirmed SARS-CoV-2 infections in the United States from February 12 to March 28, 2020. Data regards 7,162 subjects with completed case information and revealed a prevalence rate for diabetes of 6%, 24%, and 32%, for non-hospitalized, hospitalized but not requiring intensive care unit (I.C.U.) admission vs hospitalized in the I.C.U., respectively (20). In Italy, a diabetes prevalence of 17% was reported in patients admitted to intensive care units for severe COVID-19 (16). Recent data confirmed the findings mentioned above, outlining a diabetes prevalence of 18.3% in English people with severe COVID- 19 requiring critical care treatment (high dependency unit or intensive care unit) (21). Other study showed a significant prevalence of diabetes (30.05% vs 19.57%) in dead patients compared to survivors in ICU wards in Spain (22). Moreover, Du and colleagues reported a prevalence of 35.3% of diabetes in Wuhan’s ICU patients (23). In summary, diabetes resulted associated with a dramatic increase in mortality (OR 3.62; 95% CI 2.11–6.2; p<0.0001) and emerged as an independent risk factor even after correcting for age, race, sex, obesity, and hypertension (24).

Secondly, prevalence rates of diabetes (31.8% vs 5.4%) and obesity (39.8% vs 14.5%) were more significant in the hospitalized vs non-hospitalized subgroups, respectively (12). Additionally, a Body Mass Index (B.M.I.) >40 was among the risk factors most predictive of the need for hospitalization (OR 6.2, 95% CI, 4.2-9.3) (12). In addition, obesity represented by B.M.I. has still increased the mortality (obesity class I HR 1.23; obesity class II HR 1.81) (25). Severe obesity (BMI ≥ 35 kg/m2), increasing age, and male sex are independently associated with mortality and need for intubation and increasing oxygen requirements during hospitalization (26). An analysis of 124 consecutive I.C.U. admissions in a single center in Lille, France from February 27 to April 5, 2020, revealed higher obesity rates and severe obesity among SARS-CoV-2 patients, relative to historical no SARS-CoV-2 controls. In this observational study, the frequency of obesity was 47.5% compared to 25.8% in a control group of I.C.U. individuals with the non-SARS-CoV-2 illness. In subjects affected by obesity, the requirement for intubation and mechanical ventilation was higher (27).

Moreover, obesity increases the risk of hypoventilation syndrome in I.C.U. patients and it can lead to respiratory failure when ARDS is present (28–33). The likely mechanisms that explain these effects are poorly understood, but mechanical and inflammatory factors might contribute. At the same time, we have shown that obesity (especially the visceral type) is associated with low levels of adiponectin, which could be the link to explain the increased cardiovascular risk in patients who present the association between obesity and COVID-19 (34–36). Finally, according to Crouse et al., 74% of diabetic patients were obese, which may have contributed further to the increased risk observed in this population (24). Given the known epidemiological relationship above mentioned, both conditions’ coexistence can be considerate a “cumulative” risk factor for disease severity.

## Diabetes as a risk factor of worst outcomes

In general, people with diabetes are at higher risk of developing complications because of infection, particularly viral one. The differences in response are likely the result of the degree of viral load, host immune response, age of the patient, and presence of comorbidities. The higher risk of mortality and complications among people with diabetes was similar in the two other recent coronavirus outbreaks, the SARS and the Middle East respiratory syndrome (MERS) (37). Type 2 diabetes is associated with low-grade chronic inflammation induced by the excessive visceral adipose tissue. This inflammatory condition affects the homeostatic glucose regulation and peripheral insulin sensitivity. Chronic hyperglycemia and inflammation can determine an abnormal and ineffective immune response (37).

Diabetic patients with COVID-19 are at higher risk of being in an excessively hypercoagulable state and uncontrolled inflammation responses, which may contribute to a poorer outcome (38).

In particular, a known history of diabetes and a fasting plasma glucose ≥ 7.0 mmol/l before steroid treatment were independent predictors of death with an increased risk (odds ratio) of 3.0 and 3.3, respectively (39).

In a recent study on 132 patients with type 2 diabetes, a negative linear correlation between SaO2 and HbA1c was found. At the same time, there was a positive linear correlation between serum ferritin, C-Reactive Protein, Fibrinogen, and Erythro-Sedimentation Rate levels and HbA1c, especially with HbA1c ≥ 7.5% (40).

Developing data also suggest that COVID-19 patients with diabetes are more often associated with severe or critical disease with a percentage ranges from 14-32% in different studies (41–45).

Wu et al. (46) reported a hazard ratio (H.R.) of 2.34 (95% CI, 1.35 to 4.05; p=0.002) for the acute respiratory syndrome (ARDS) in a sample of 201 diabetic patients with COVID-19.

However, in their meta-analysis (n=46,248), Yang et al. reported that the odds ratio (OR) of severe COVID-19 was not significantly higher in people with diabetes (OR, 2.07; 95% CI, 0.89 to 4.82), unlike hypertension (OR, 2.36; 95% CI, 1.46 to 3.83) (9).

The lower prevalence of diabetes might explain this finding, and the lower age of the populations studied. Another meta-analysis of 9 studies from China (n=1936) by Chen et al. found a significant association between COVID-19 severity and diabetes (OR, 2.67, 95% CI; 1.91 to 3.74; p<0.01) (47). Curiously, the prevalence of non-survivors was also higher in diabetic subjects with COVID-19 (42, 48, 49). Also, a summary report of 44,672 patients of COVID-19 from the Chinese Center for Disease Control and Prevention reported a case fatality rate (C.F.R.) of 2.3% (1023 deaths among 44,672 confirmed cases). In any case, the C.F.R. was as high as 7.3% in patients with diabetes and 6.0% in hypertension (50).

However, conflicting data exist. Indeed, in univariate analysis of 191 patients with COVID-19, Zhou et al. (48) found that diabetes had an OR of 2.85 (95% CI, 1.35 to 6.05; p<0.001) for in-hospital mortality. Nevertheless, the association of diabetes and mortality was no longer significant after a multivariate regression analysis showing an independent and positive relationship with age, SOFA score, and D-Dimer levels.

In 174 consecutive patients confirmed with COVID-19, data between diabetics and non-diabetics were compared according to signs and symptoms, laboratory findings, and chest computed tomography. Patients with diabetes had no significant differences in gender and age, less fever (59.5% vs 83.2%), more nausea and vomiting (16.7% vs 0%), and higher mortality (16.7% vs 0%) in comparison with patients without diabetes.

The absolute counts of neutrophils and some inflammation-related biomarkers’ serum levels are much higher than those without diabetes, such as IL-6, serum ferritin, E.S.R., C.R.P, and D-dimer. Beyond that, the absolute count of lymphocytes, red blood cells, and haemoglobin level was significantly lower in the diabetes group than in the non-diabetes group. It is crucial to outline that the levels of enzymes such as L.D.H., α-hydroxybutyrate-dehydrogenase, A.L.T., and G-GT, indicating the injury of myocardium, kidney, and liver were even higher in patients with diabetes than patients without diabetes. These data provide a clue that the injure of organs was much more severe in the diabetics group than those without diabetes (38).

Finally, even when chest computed tomography (C.T.) imaging of COVID-19 patients with or without diabetes was compared, the latter showed more severe pathological changes than the former. To confirm this, the diabetes group presented higher C.T. imaging scores in comparison with the non-diabetes group (38).

### Proposal of Pathogenetic Mechanisms

There are several specific factors and mechanisms by which diabetes predisposes to infections in general and may increase susceptibility or risk and severity of SARS-CoV-2 disease. Potential mechanisms that may enhance the susceptibility for COVID-19 diabetics include the role of hyperglycemia, higher affinity cellular binding, and efficient virus entry, decreased viral clearance, diminished T cell function, increased susceptibility to hyper-inflammation, cytokine storm syndrome, and presence of CVD (51).

## The Role of Hyperglycemia

The susceptibility to SARS appears to be primarily dependent on the spike’s affinity to bind host ACE2 receptors (ACE2r) in target tissues in the initial viral attachment step (52).

ACE2r has been confirmed recently as the SARS-CoV-2 internalization receptor causing COVID- 19, in concert with the host’s TMPRSS2 membrane protease that primes the spike S protein of the virus to facilitate its cell entry (53).

A possible explanation for a link between hyperglycemia and ACE2r levels in the severity of COVID- 19 disease could be the potential changes in glycosylation of the ACE2r and glycosylation of the viral spike protein.

Both possibly resulting from uncontrolled hyperglycemia may alter the viral spike protein binding to ACE2r and the degree of the immune response to the virus (54). Elevated glycemia levels can directly increase glucose concentrations in airway secretion (55). According to Brufsky, potentially, in uncontrolled hyperglycemia, high and aberrantly glycosylated ACE2r in the lung, nasal airways, tongue, and oropharynx could also serve as increased SARS-CoV-2 viral binding sites, thus leading to a higher trend to COVID-19 infection and a more severe form of the disease (54). This indicates the presence of stress hyperglycemia (i.e., a temporary increase in blood sugar in patients with A1C <6.5% after acute illness or surgery) (56), which may have a worse outcome in acute illness, compared to a previously diagnosed diabetes. Given that stress hyperglycemia patients have observed similar worse results in the previous meta-analysis, this finding is not unexpected (57, 58). Stress hyperglycemia was one of the poor prognostic factors and had been associated with a significant increase in respiratory failure and death in subjects with SARS (59).

Glycemic control could reduce levels of glycosylated ACE2r target in the lung. In this way, the number of glycosylated viral binding sites decreases, possibly ameliorate inflammation and symptoms of COVID-19 disease (54). ACE2r is expressed not only in type I and II alveolar epithelial cells in the lungs and upper respiratory tract, but also in several other locations like the heart, endothelium, renal tubular epithelium, intestinal epithelium, and pancreas (54).

Within the pancreas, ACE2 expression has been described in acinar cells and within subsets of islet cells. In preclinical studies, gain and loss of ACE2 function reveal physiological and pharmacological roles for ACE2, both dependent and independent of Angiotensin (1-7), which may antagonize the actions of angiotensin II in glucose control and cell function, renal physiology, blood pressure, atherosclerosis and amelioration of experimental diabetes (60, 61).

An acute viral respiratory infection has been linked to the rapid development of transient insulin resistance, both in otherwise healthy euglycemic normal weight or overweight individuals (62).

This link suggested a mechanism of transient hyperglycemia induced by a temporary inflammation of the pancreas’ islet cells by SARS-CoV through SARS-CoV binding to the ACE2r present on islet cells, resulting in a transient insulin-dependent diabetes mellitus, which resolved with the resolution of disease (63).

Hyperglycemia may also affect pulmonary function, such that it is exacerbated by influenza virus-induced respiratory dysfunction in patients with diabetes. In animal models of disease, diabetes is associated with numerous structural changes to the lung such as augmented permeability of the vasculature and a collapsed alveolar epithelium (64).

Until a short time ago, whether D.M. was causally linked to ACE2r expression levels in the lung in humans was unknown. Rao et al., by a phenome-wide Mendelian randomization study, explored diseases that may be causally linked to increased ACE2 expression in the lung, showing that diabetes was causally associated with increased lung ACE2 expression (65).

Despite a possible role of TMPRSS2 in the viral pathogenicity, little information is available about the regulation or dysregulation of TMPRSS2 expression or activity by glucose in the context of experimental or clinical diabetes. In patients with diabetes, higher circulating glucose levels will result in a higher percentage of glycated haemoglobin. SARS-CoV-2 surface proteins seem to bind to and potentially impair the heme molecule within red blood cells. In this way, a separation of iron from the molecule to form a porphyrin occurs, determining in red blood cells less oxygen and carbon dioxide carriage, thereby generating cell death and intense inflammation in the lung.

Because patients with diabetes and older people have more glycated haemoglobin, they may be preferentially affected by SARS-CoV-2 binding and dissociation of iron from heme to form porphyrins, and another receptor (CD147 or basigin) might be involved (66).

## Impaired T-Cell Function and Increased Susceptibility to Hyperinflammation

The activation of proinflammatory cytokines or chemokines causes infected cells apoptosis or necrosis and triggers inflammatory responses, which leads to the recruitment of inflammatory cells. Through interferon-gamma (I.F.N) production, CD4 T helper (Th1) cells are involved in regulating antigen presentation against intracellular pathogens such as CoV. The recruitment of neutrophils and macrophages is induced by Th17 cells by producing interleukin-17 (IL-17), IL-21, and IL-22 (67).

SARS-CoV-2 increases apoptosis of lymphocytes (CD3, CD4, and CD8 T cells) and infects circulating immune cells leading to lymphocytopenia.

Lower T cell function diminishes the inhibition of innate immune system resulting in the secretion of high amounts of inflammatory cytokines. This phenomenon is called “cytokine storm” (68). Neutrophil chemotaxis, the intracellular killing of microbes, and phagocytosis were inhibited by diabetes. In the beginning, a delay in the activation of Th1 cell-mediated immunity and a late hyper-inflammatory response is often observed in diabetics (69).

In line with this evidence, in patients with COVID-19, the number of CD4+ and CD8+ T cells are low. However, a higher proportion of highly pro-inflammatory Th17 CD4+ T cells, along with elevated cytokine levels was present. It is possible to speculate that patients with D.M. may have weakened anti-viral I.F.N. responses, and the delayed activation of Th1/Th17 may accentuate inflammatory responses (51).

Several cytokines are increased in COVID-19 infection. At baseline, cytokines such as TNF, IL-1, and IL-6 are more active in diabetics and subjects affected by obesity. Therefore, it is believed that SARS-CoV-2 infection may enhance the cytokine response of such patients, thereby exacerbating the cytokine storm that seems to cause multiple organ failure in COVID-19 (33, 70).

The baseline proinflammatory state found in diabetes and obesity may serve to exacerbate this. Diabetes occurs in part because the increase of activated innate immune cells in metabolic tissues leads to the overproduction of inflammatory mediators, especially IL-1β and TNFα, which can promote systemic insulin resistance and β cell damage (9).

## Therapeutical Considerations

### Metformin

In preclinical studies, metformin exerts anti-inflammatory action and reduces circulating biomarkers of inflammation in people with T2D (71). However, there is scant information about the immunomodulatory actions of metformin in the context of coronavirus infection. Recent data showed that the use of metformin significantly reduced the odds of dying. People tested positive who were taking metformin had an 11% risk of dying, which was the same as the general COVID-19 population and dramatically lower than 24% mortality of subjects with diabetes not taking metformin. Interestingly, even after correcting for insulin use, age, race, sex, obese status, and hypertension status, the likelihood of death in subjects with T2D taking metformin was significantly lower than those who did not take metformin (OR 0.33; 95% CI 0.13–0.84; p=0.0210) (24).

Anyway, metformin should be used with caution in unstable hospitalized patients and should be discontinued in people with concomitant sepsis or severe impairment of hepatic and renal function (72). If vomiting or poor oral intake occurs, metformin may also be stopped. Due to the level of blood sugar, the dosage of sulfonylureas and insulin may have to be changed (6).

## GLP-1R agonists

GLP-1R agonists exert broad anti-inflammatory actions in animals with experimental inflammation and reduce biomarkers of systemic inflammation in human subjects with T2D and people with obesity (73). It is well known that the most severe form of COVID-19 is the Acute Respiratory Distress Syndrome characterized by the highest levels of inflammatory cytokines known as “Cytokine Storm” which hurts alveolar epithelial cells in the lung, inactivates pulmonary surfactant resulting in the formation of the hyaline membrane and lung parenchyma breakdown.

Different preclinical studies showed that GLP-1R agonists attenuate pulmonary inflammation, reduce cytokine production and preserve lung function in mice and rats with experimental lung injury (74, 75) through the stimulation of pulmonary vasodilator like atrial natriuretic peptide and facilitation of surfactant protein A. GLP-1R agonists exert the repression of the proinflammatory cytokine, the stimulation of eNOS/sGC/PKG signaling and cause the inactivation of the NF-κB signaling. In addition, in the LPS-induced acute lung inflammatory injury mouse model, liraglutide attenuates the expression of key inflammasome components, such as thioredoxin-interacting protein (TxNIP) significantly increased after the administration of lipopolysaccharide (L.P.S.) along with cytokines and chemokine genes (73).

These beneficial effects could identify GLP-1-based drugs as fundamental tools for treating COVID-19 patients with or without diabetes. Although GLP-1 safely lowers blood glucose of ventilated patients with critical illness in short term studies, there is still insufficient safety and experience in using GLP-1R agonists in critically ill patients, and it is impossible to make treatment recommendations for the use of these drugs in new coronavirus infection (76).

In our opinion, GLP-1R agonists must not be suspended if previously prescribed. Moreover, further studies need to evaluate the beneficial effect as the first prescription in diabetes patients with COVID- 19.

## Dipeptidyl Peptidase-4 (DPP4)

Recently, coronavirus’s relationship to cellular type-II transmembrane protein DPP4, also known as adenosine deaminase complexing protein 2 or cluster of differentiation 26 (CD26), has generated a great interest. Just as ACE-2 is the receptor for SARS CoV and SARS CoV2, DPP4 acts as the receptor for MERS-CoV. Whether the use of DPP4 inhibitors (DPP4i) can reduce MERS-CoV’s viral entry has aroused great interest. In vitro study, sitagliptin, vildagliptin, and saxagliptin did not prevent the coronavirus from entering the cell (74).

Briefly, DPP4i seem to increase inflammation in type 2 diabetes *via* catalytic and noncatalytic mechanisms. It is crucial to outline that the enzymatic activity of DPP4 causes the cleavage and may affect the function of several chemokines, cytokines, and growth factors (37).

However, in patients with type 2 diabetes, the effects of DPP4 inhibition on the immune response is controversial and not completely understood. A meta-analysis showed that DPP4 inhibitor treatment does not increase significantly upper respiratory tract infections. Compared with placebo or active comparator treatment, risks of respiratory infection for DPP4i were all comparable. The initiation of a DPP4 inhibitor was not associated with an increased risk of respiratory tract infections. On the contrary, anti-inflammatory and anti-adipogenic effects have been related to the use of DPP4 inhibitors and GLP-1 receptor analogues (37). In fact, gliptins can preserve endothelial function by their reported anti-inflammatory, anti-oxidant, and potentially protective effects on the vascular system, which are beneficial aspects in the fight against COVID-19 (77).

## Insulin

Insulin exerts anti-inflammatory actions in humans and reduces biomarkers of inflammations in hospitalized individuals with a critical illness. Among available agents for the treatment of acute disease complicated by diabetes, insulin has been the most extensively used agent in human subjects with bacterial or viral infections and in hospitalized critically ill patients. Most hospitalized patients with COVID-19, especially those with respiratory distress, would require insulin.

However, there is little information surrounding the potential benefits or risks of insulin in the context of acute coronavirus infection (75, 76). On the other hand, one should consider the importance of maintaining a reasonable glycemic control in this patient. This statement may be remarkably faithful to the light of recent evidence that showed, in a sample size of fifty-nine patients, that insulin infusion is beneficial for achieving glycemic targets and improving outcomes in patients with COVID-19 (78).

## SGLT2-Inhibitors

Sodium-glucose cotransporter-2 inhibitors (SGLT2i) are demonstrated to have cardiovascular and renal benefits in addition to its anti-diabetic effects (79). Clinical experiments showed that the combined use of SGLT2i and ACEI/ARB significantly increases intrarenal ACE2 expression, which may be closely related to improving cardiac and renal function (80). However, the increased ACE2 may be detrimental to patients infected with the coronavirus infection 2019 (COVID-19), which is found to invades cells *via* the entry receptor of ACE2. Besides, SGLT2i induced natriuretic effect may also increase the risk of acute kidney injury and affect hemodynamic stability during systemic infection (81). SGLT2i have been reported to prevent the release of various proinflammatory cytokines such as IL 6. Besides, SGLT2i lead to an increase in the ACE-2 levels, which leads to greater production of the angiotensin 17, which is a potent vasodilator, anti-oxidant, and anti-fibrotic, which helps in the prevention of acute respiratory distress syndrome (ARDS) and alleviating cytokine storm (82). This may have a putative role in COVID-19 patients with myocarditis and adverse cardiac remodeling. SGLT2i also downregulates the expression of inflammatory genes and reduces oxidative stress, leading to cardiorenal dysfunction’s amelioration (83).

## A.C.E. Inhibitors/A.R.B.s

In the absence of further evidence of risk or benefit, the American Heart Association, the American College of Cardiology, and the American Society of Hypertension have recommended that people continue treatment with their usual antihypertensive therapy ACE-inhibitors or ARBs (84). According to a recent study (13), the use of A.C.E. inhibitors in COVID-19 patients, was associated with lower mortality after hospital discharge (no association was found for the use of A.R.B.s). Whether there is an immunosuppressive state, the presence or absence of hyperlipidemia or diabetes mellitus and the race or ethnic group were not independent predictors of death in the hospital.

## Statins

Besides their lipid-lowering activity, statins exert pleiotropic effects on inflammation and oxidative stress, contributing to their beneficial impact on cardiovascular diseases. Statins modulate the immune response at different levels, including immune cell adhesion and migration, antigen presentation, and cytokine production.

Statins also interfere with ACE2 signaling. After initial entry through ACE2, SARS-CoV-2 down-regulates ACE2 expression, possibly facilitating the initial infiltration by innate immunity cells and causing an unopposed angiotensin II accumulation, leading to organ injury.

Furthermore, in COVID-19 infection, statins’ lipid-lowering action could treat the hyperlipidemia associated with the use of protease-inhibitor-based antiretroviral and immunosuppressive drugs. The hepatic isoenzyme CYP3A4 metabolizes simvastatin and, to a lesser extent, atorvastatin. Concomitant administration of CYP3A4 inhibitors such as ritonavir and cobicistat, currently used in COVID-19, could increase the risk of muscle and liver toxicity.

Therefore, starting with a lower dose of statin and monitoring creatine kinase and transaminases would be advisable (85).

Moreover, the same problem occurs with azithromycin. The same study above mentioned (13) pointed out the statins as an independent protective factor for survival to hospital discharge.

## Clinical Consideration

Data reported in our review support the notion that diabetes should be considered as a risk factor not only for increased susceptibility to infection but also for a rapid progression and bad prognosis of COVID-19. Therefore, according to pathophysiological consideration, people with diabetes should be paid more intensive attention to the importance of glycosylation of both the viral spike protein and ACE2r.

This consideration argues for better glycemic control in patients with hyperglycemia at hospital admission. Pre-diabetes and diabetes are potential mechanisms to slow the spread of COVID-19 and reduce symptoms and improve outcomes. We know that blood glucose control is essential for patients with COVID-19 and those without the disease. Nevertheless, hyperglycemia is still a powerful predictor of the prognosis of hospitalized Covid-19 patients. Besides, Covid-19 patients with hyperglycemia, compared with subjects normoglycemia, showed a higher cumulative incidence of serious diseases. In addition, optimal blood glucose control mediated by insulin infusion can improve the prognosis of hospitalized Covid-19 and hyperglycemic patients (78). Patients with mild infections, and regular oral doses can continue routine hypoglycemic drugs. However, in today’s world, with an unprecedented pandemic, the treatment of diabetes presents challenges. In most places, people are confined to “closed areas”, exercise opportunities are limited, and they cannot take regular walks and visit gyms or swimming pools. Due to the unpredictability and social nature of the disease, mental stress is also great. Do not move as well as social immobility. Alterations in the daily routine affect dietary intake as well. Stress could lead to inappropriate eating. All these factors may lead to uncontrolled glycemia or worsening status of comorbid diseases (e.g., hypertension) and predispose the patients to complications like other types of infections, hyperosmolar coma, ketoacidosis, and even acute cardiovascular or cerebrovascular events (6).

In this sense, telemedicine could come to assistance. In a Cochrane review, Flodgren and colleagues analyzing 21 randomized controlled trials of 2768 patients with diabetes showed that a significant reduction of HBA1c by -0.31% (p < 0.001) was present in patients on telemedicine in comparison with controls (86). In a recent systematic review and meta-analysis which included patients with type 2 diabetes mellitus and type 1 diabetes mellitus Timpel and colleagues showed that there was a mean reduction in HbA1c levels in type 1 (-0.12 to -0.86%) and type 2 diabetes patients (-0.01% to -1.13%) included in the telemedicine intervention group (87).

According to this view, it is possible to summarize measures for good health in patients with diabetes:

- Diabetics need to maintain a regular diet.
- According to the American Diabetes Association (88), exercises should be continued at home.
- Physical activity improves blood glucose control in type 2 diabetes (aerobic exercise increases muscle glucose uptake up to fivefold through insulin-independent mechanisms), reduces cardiovascular risk factors, contributes to weight loss. Adults affected by type 2 diabetes should ideally perform both aerobic and resistance exercise training for optimal glycemic and health outcomes (89).
  Structured lifestyle interventions including at least 150 min/week of physical activity and dietary changes are recommended to promote weight loss of 5%-7% and to prevent or delay the onset of type 2 diabetes in people at high risk and with pre-diabetes.
- Regular intake of ACE2-inhibitors and other antihypertensive and anti-diabetic drugs (including GLP1-R agonists) and, if necessary, insulin is important and should be emphasized for the best blood pressure glucose control.
- Telemedicine can be beneficial in these times. Patients can consult their physicians *via* telemedicine, who can give appropriate advice about treatment (90).

In conclusion, COVID-19, due to its heterogeneity, can be considered a systemic disease as it is more than a single-organ disease (4). The association with diabetes makes the whole condition at a higher risk of poor outcomes. For this reason, from now on, diabetes care has to include a careful, holistic evaluation of all the patients affected by COVID-19.

## Data Availability Statement

The original contributions presented in the study are included in the article/supplementary material. Further inquiries can be directed to the corresponding author.

## Author Contributions

KP, MV, and MR have collected the various studies. CA and SC were responsible for revising the article critically. All authors contributed to the article and approved the submitted version.

## Conflict of Interest

The authors declare that the research was conducted in the absence of any commercial or financial relationships that could be construed as a potential conflict of interest.
